# Glucose‐induced oxidative stress and accelerated aging in endothelial cells are mediated by the depletion of mitochondrial SIRTs

**DOI:** 10.14814/phy2.14331

**Published:** 2020-02-05

**Authors:** Jieting Liu, Shali Chen, Saumik Biswas, Niharika Nagrani, Yanhui Chu, Subrata Chakrabarti, Biao Feng

**Affiliations:** ^1^ Department of Pathology and Laboratory Medicine Western University London ON Canada; ^2^ Mudanjiang Medical University Heilongjiang PR China

**Keywords:** aging, diabetic complications, endothelial cells, miRNAs, SIRT

## Abstract

Diabetic complications cause significant morbidity and mortality. Dysfunction of vascular endothelial cells (ECs), caused by oxidative stress, is a main mechanism of cellular damage. Oxidative stress accelerates EC senescence and DNA damage. In this study, we examined the role of mitochondrial sirtuins (SIRTs) in glucose‐induced oxidative stress, EC senescence, and their regulation by miRNAs. Human retinal microvascular endothelial cells (HRECs) were exposed to 5 mmol/L (normoglycemia; NG) or 25 mmol/L glucose (hyperglycemia; HG) with or without transfection of miRNA antagomirs (miRNA‐1, miRNA‐19b, and miRNA‐320; specific SIRT‐targeting miRNAs). Expressions of SIRT3, 4 and 5 and their targeting miRNAs were examined using qRT‐PCR and ELISAs were used to study SIRT proteins. Cellular senescence was investigated using senescence‐associated β‐gal stain; while, oxidative stress and mitochondrial alterations were examined using 8‐OHdG staining and cytochrome B expressions, respectively. A streptozotocin‐induced diabetic mouse model was also used and animal retinas and hearts were collected at 2 months of diabetes. In HRECs, HG downregulated the mRNAs of SIRTs, while SIRT‐targeting miRNAs were upregulated. ELISA analyses confirmed such downregulation of SIRTs at the protein level. HG additionally caused early senescence, endothelial‐to‐mesenchymal transition and oxidative DNA damage in ECs. These changes were prevented by the transfection of specific miRNA antagomirs and by resveratrol. Retinal and cardiac tissues from diabetic mice also showed similar reductions of mitochondrial SIRTs. Collectively, these findings demonstrate a novel mechanism in which mitochondrial SIRTs regulate glucose‐induced cellular aging through oxidative stress and how these SIRTs are regulated by specific miRNAs. Identifying such mechanisms may lead to the discovery of novel treatments for diabetic complications.

## Graphical abstract

We examined the role of mitochondrial sirtuins (SIRTs) in glucose‐induced oxidative stress, EC senescence and their regulation by miRNAs by using RT‐qPCR, ELISA, Western blot, senescence‐associated β‐gal stain and 8‐OHdG immunofluorescence staining. Our results showed a novel mechanism in which mitochondrial SIRTs regulate glucose‐induced cellular aging through oxidative stress and how these SIRTs are regulated by specific miRNAs. Identifying such mechanisms may lead to novel treatments for diabetic complications.

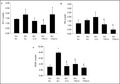

## 1 INTRODUCTION

In diabetes, chronic hyperglycemia causes a large variety of complications that can affect the peripheral nerves, eyes, kidneys, and heart (Alam, Asghar, Azmi, & Malik, 2014). Such damages are initiated through a series of biochemical alterations at the cellular level, which subsequently lead to micro‐ and macrovascular dysfunctions (Walther et al., 2015). In particular, endothelial cells (EC) in the retinal vasculature are primary targets of glucose‐induced damage in diabetic retinopathy (DR) (Fiori et al., 2018). Endothelial dysfunction (ED) is a characteristic feature of DR and is a key event in the early development and progression of the disease (Ao, Liu, Li, & Lu, 2019; Zhang et al., 2017).

In diabetes, increases in oxidative stress disrupt EC homeostasis (Kimura et al., 2017). In response to high glucose levels, proton gradients in the mitochondrial electron transport chain (ETC) are elevated, which initiate oxidative stress and the overproduction of superoxides (Blecha et al., 2017). Oxidative stress changes cellular transcriptional machinery leading to increased production of vasoactive factors (endothelin‐1 [ET‐1] and vascular endothelial growth factor [VEGF]), growth factors, and cytokines that ultimately cause alterations of organ hemodynamics, increased production of extracellular matrix (ECM) proteins (i.e., fibronectin; FN) (Abd El‐Kader & Saiem Al‐Dahr, 2016; Iwona, 2016; Ohshiro et al., 2006; Xu, Chiu, Feng, Chen, & Chakrabarti, 2008), structural alterations (i.e., endothelial‐to‐mesenchymal transition), and neovascularization. Interestingly, all changes seen in chronic diabetic complications are also seen in the normal aging process; however, these processes are accelerated in diabetes (Mortuza, Chen, Feng, Sen, & Chakrabarti, 2013).

Silent information regulator proteins or sirtuins (SIRTs) are class III histone deacetylases and known to cause epigenetic gene silencing (Dali‐Youcef et al., 2007). As they were found to increase lifespan in yeast, they have been cited as “longevity genes”. SIRTs demonstrate multiple functions and play important roles in aging, calorie restriction, inflammation, DNA damage, and metabolic regulation (Dali‐Youcef et al., 2007).

In mammals, SIRTs represent a family of seven members (SIRT1 through SIRT7) and can be localized in the cell nucleus (SIRT1, 2, 6, 7), cytoplasm (SIRT1, 2), and in the mitochondria (SIRT3, 4, 5). Mitochondrial SIRTs maintain mitochondrial integrity via maintenance of mitochondrial dynamics and signaling pathways (Singh et al., 2018). The functional capabilities of these SIRTs allow cells to respond to oxidative stress and they further play important roles in nutrient stress and cell survival (Parihar, Solanki, Mansuri, & Parihar, 2015). Alterations of mitochondrial SIRTs have been demonstrated in various malignancies, metabolic diseases, aging, and in neurodegeneration (Trostchansky, Quijano, Yadav, Kelley, & Cassina, 2016).

We have previously demonstrated glucose‐induced downregulation of SIRT1 using both in vivo and in vitro model systems. We have also shown that such downregulation accelerates glucose‐induced aging in ECs and also leads to increased production of multiple ECM proteins and vasoactive factors in diabetes (Mortuza et al., 2013). However, since the mitochondrion is the main site for the generation of oxidative stress, it is imperative to investigate mitochondrial SIRTs in this context. Hence, we examined three mitochondrial SIRTs, namely SIRT3, SIRT4, and SIRT5. SIRTs 3–5 are localized in the mitochondria and regulate the activities of metabolic enzymes and moderate oxidative stress in this organelle (Bause & Haigis, 2013; Lang et al., 2017; Schlicker et al., 2008). We hypothesized that glucose‐induced oxidative stress and EC damage are mediated through alterations of mitochondrial SIRTs and that specific miRNAs regulate such altered expressions. We specifically examined glucose‐induced alterations of these molecules in vivo and in vitro. We further expanded these studies to examine possible microRNA‐mediated regulations of these SIRTs, as well as their roles in oxidative DNA damage and the production of downstream effector molecules.

## 2 MATERIAL AND METHODS

### 2.1 Cell culture and treatments

All reagents were purchased from Sigma (Oakville, Ontario, Canada) unless specified. Human retinal microvascular endothelial cells (HRECs) were obtained from Olaf Pharmaceuticals. HRECs were grown in endothelial cell basal medium 2 (EBM‐2, Lonza) supplemented with 5% fetal bovine serum, endothelial cell growth supplement (Bio‐Whittaker), and 100 μg/ml penicillin/streptomycin. Cells were plated at a density of 1 × 10^5^ cells/ml. All cells were maintained in a humidified atmosphere containing 5% CO_2_ at 37°C incubation. The cells were cultured in six‐well plates (Corning) and were treated with various concentrations of D‐glucose (5 mmol/L, representing normal glucose [NG], or 25 mmol/L, representing high glucose [HG]) or 25 mmol/L L‐glucose (osmotic control) for 48 hr. As for the miRNA‐based experiments, cells were transfected with either a miRIDIAN miRNA antagomir for miR‐1, miR‐19b, miR‐320a or a negative control miRNA (100 nM for each miRNA) (Thermo Fisher Scientific) using the transfection reagent Lipofectamine 2000 (Invitrogen). After transfection for 24 hr, the cells were changed into serum‐free media and treated with HG for another 48 hr. For the resveratrol experiments, cells were treated with 10 µM resveratrol (Sigma) dissolved in ethanol and cultured in HG media for 48 hr (Chen et al., 2010; Feng et al., 2016; Mortuza et al., 2013).

### 2.2 Animals

All animals were kept in our university facility according to the Guiding Principles in the Care and Use of Animals. The experiments were approved by Western University and Animal Care and Veterinary Services. All experiments conform to the Guide for Care and Use of Laboratory Animals published by the National Insitutes of Health (NIH Publication 85‐23, revised in 1996).

Male C57BL/6J mice (8–10 weeks, 23–25 g) were obtained from the Charles River Colony (Wilmington, MA) and kept in our university facility for 1 week. These mice were then randomly divided into control and diabetic groups. Streptozotocin (STZ; Sigma‐Aldrich) was obtained and prepared in cold 0.1 M citrate buffer (pH 4.5) prior to injection. As previously described, diabetes was induced by intraperitoneal injections of STZ (50 mg kg^−1^ day^−1^ in citrate buffer, pH 4.5) for 5 consecutive days (Feng et al., 2016). The control group received an equal volume of citrate buffer. Using the Freestyle Freedom Lite blood glucose monitoring system (Abbott Diabetes Care), diabetes was diagnosed when blood glucose levels were greater than 16.7 mmol/L (300 mg/dl) on two consecutive tests. The animals were fed on a standard rodent diet with water ad libitum and mice were euthanized (via carbon dioxide inhalation) after 2 months, following the development of diabetes (*n* = 8/group). Retinal and cardiac tissues were collected for gene and protein expression analyses. All tissues were stored at −80°C immediately after collection.

### 2.3 mRNA analysis with real‐time qRT‐PCR

Total cellular RNA was isolated using the TRIZOL™ (Invitrogen) reagent, as established by our laboratory (Chen et al., 2010; Feng et al., 2016; Mortuza et al., 2013). Briefly, RNA was extracted with chloroform followed by centrifugation to separate the sample into aqueous and organic phases. The RNA was recovered from the aqueous phase by isopropyl alcohol precipitation and the pellet was ultimately dissolved in DEPC water. RNA concentration was assessed on a spectrophotometer (Gene Quant‐Pharmacia Biotech). cDNA was made using High Capacity cDNA Reverse Transcription kit (Applied Biosystems) according to the manufacturer's instructions. Real‐time qRT‐PCR was performed in the LightCycler™ (Roche Diagnostics) to quantify the mRNA expressions of SIRT3, SIRT4, and SIRT5. All the primers were either ordered or custom made from Takara Bio USA Inc. (Table 1). For a final reaction volume of 20 μl, the following reagents were added: 7 μl of H_2_O, 10 μl of SYBR (Sigma‐Aldrich), 1 μl forward and reverse primer, and 1 μl of cDNA. The data were analyzed using the standard curve method and normalized to β‐actin mRNA to account for differences in reverse‐transcription efficiencies and the amount of template in the reaction mixtures.

**Table 1:** Oligonucleotide sequences for qRT‐PCR

Gene	Sequence (5′ → 3′)
SIRT3 (human)	CATGAGCTGCAGTGACTGGT
	GAGCTTGCCGTTCAACTAGG
SIRT4 (human)	CAGCAAGTCCTCCTCTGGAC
	CCAGCCTACGAAGTTTCTCG
SIRT5 (human)	CCAGATTGTCCCAAGTCGAT
	CTGAAGGTCGGAACACCACT
SIRT3 (mouse)	AGGTGGAGGAAGCAGTGAGA
	GCTTGGGGTTGTGAAAGAAA
SIRT4 (mouse)	TCCAAAGGCTGGAAATGAAC
	GCGACACAGCTACTCCATCA
SIRT5 (mouse)	CATCACCCAGAACATTGACG
	CAGGATCCAGGTTTTCTCCA
β‐actin (human/mouse)	CATCGTACTCCTGCTTGCTG
	CCTCTATGCCAACACAGTGC
CD31(human)	AGACAACCCCACTGAAGACGTCG
	CCTCTCCAGACTCCACCACCTTAC
VE‐cadherin (human)	CTACCAGCCCAAAGTGTGTG
	GTGTTATCGTGATTATCCGTGA
SM22 (human)	GGCAGGCCCCAGTAAAGAAG
	TGCCAGCCCACCCAGATT
Vimentin (human)	GGCTCAGATTCAGGAACAGC
	CTGAATCTCATCCTGCAGGC
ET‐1 (human)	GAAACCCACTCCCAGTCCAC
	CCAGGTGGCAGAAGTAGACA
FN (human)	GATAAATCAACAGTGGGAGC
	CCCAGATCATGGAGTCTTTA
VEGF (human)	GAACTTTCTGCTGTCTTGGG
	CTTCGTGATGATTCTGCCCT
CytB (human)	TCACCAGACGCCTCAACCGC
	GCCTCGCCCGATGTGTAGGA

### 2.4 Senescence‐associated β‐galactosidase (SA β‐gal) activity

Cells were fixed with 3% formaldehyde in PBS, pH 7.4 for 5 min and incubated at 37°C overnight in staining solution [40 mM sodium citrate, pH 6.0, 1% 5‐bromo‐4‐chloro‐3‐indolyl‐β‐D‐galactopyranoside (X‐gal), 5 mM potassium ferrocyanide, 5 mM ferricyanide, 150 mM sodium chloride, and 2 mM magnesium chloride]. Following staining, nearly 200 cells were evaluated in each group and the cells were imaged using phase‐contrast microscopy (Debacq‐Chainiaux, Erusalimsky, Campisi, & Toussaint, 2009; Mortuza et al., 2013).

### 2.5 miRNA detection by real‐time qRT‐PCR

Reverse transcription was performed after isolating miRNA using a commercially available kit (Life Technologies Inc.). TaqMan™ miR‐1, miR‐19b, and miR‐320a assays (Ambion Inc.) were used for qRT‐PCR to analyze the expressions of miR‐1, miR‐19b, and miR‐320a, in accordance with the manufacturer's instructions. Normalization was performed to U6 snRNA to account for differences in reverse‐transcription efficiencies and amount of template in the reaction mixtures (Biswas et al., 2018; Feng et al., 2014; Feng, Cao, Chen, Ruiz, & Chakrabarti, 2016; Mortuza et al., 2013).

### 2.6 Detection of DNA damage by immunofluorescence

HRECs were seeded in eight‐well Nunc™ Lab‐Tek™ Chambered Coverglasses and incubated for 48 hr in the presence of glucose (5 mmol/L or 25 mmol/L). Briefly, these cells were fixed with ethanol for staining with first antibody 8‐OHdG antibody (Santa Cruz Biotehnology). After washing, the cells were then incubated with goat IgG labeled with FITC (Vector Laboratories) and the slides were mounted in Vectashield fluorescence mounting medium with 4,6‐diamidino‐2‐phenylindole (DAPI; Vector Laboratories) for nuclear staining. About 200 cells were evaluated in each group and microscopic observation was performed by an examiner unaware of the identity of the sample, using a Zeiss LSM 410 inverted laser scan microscope equipped with fluorescein, rhodamine, and DAPI filters (Carl Zeiss Canada) (Farhangkhoee, Khan, Chen, & Chakrabarti, 2006).

### 2.7 Enzyme‐linked immunosorbent assay

An ELISA was performed to measure the protein expressions of mitochondrial SIRTs using a commercially available kit (AVIVA), following the manufacturer's instructions (Chen et al., 2010).

### 2.8 Mitochondrial membrane potential (Δψm) analysis

Briefly, HRECs were treated with glucose and transfected with miRNA mimics, and after 48 hr of treatment, the cells were incubated for 10 min with 10 μM of JC‐1. These cells were then washed three times using the JC‐1 dilution buffer and fluorescence images were captured at 20× magnification using a Zeiss LSM 410 inverted laser scanning microscope (Carl Zeiss Canada), Japan. Images were analyzed with ImageJ software. Hoechst 33342 was used as a nuclear stain following the JC‐1 staining.

### 2.9 Western blotting

HRECs were exposed to glucose and transfected with miRNA mimics for 48 hr. Total proteins were prepared with RIPA buffer (Thermo Fisher) containing protease inhibitors (complete Mini Tablet; Roche). Protein concentrations were determined using Pierce™ BCA Protein Assay Kit (Thermo Scientific). A total of 30 μg of protein was resolved using SDS‐PAGE and transferred onto the PVDF membrane (Bio‐Rad), which was followed by blocking with 5% nonfat dry milk powder in TBS‐T and incubation with one of the primary antibodies: rabbit anti‐SIRT3 (1:1,000, Cell Signaling technology), mouse.

(AKL5C1) (1:1000, Santa Cruz) and mouse anti‐β‐actin antibody (1:1000, Santa Cruz) at 4°C overnight. After washing with TBS‐T, the membrane was incubated with HRP‐tagged secondary anti‐rabbit and anti‐mouse antibody (1:1000) for 1 hr at room temperature. Blots were visualized using electrochemiluminescence (Amersham Pharmacia Biotechnology). Relative band intensities were quantified by densitometry analysis using Image Lab software (Bio‐Rad).

### 2.10 Statistical analysis

Data are expressed as mean ± *SEM*. Significant differences were determined using one‐way ANOVA for multiple comparisons (followed by Tukey's post hoc) or Student's *t* test for comparing the two conditions. Differences were considered statistically significant at *p* < .05.

## 3 RESULTS

**Figure 1:**
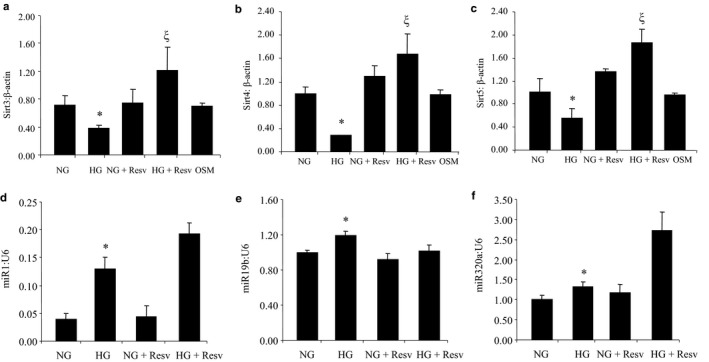
Expression of mitochondrial SIRTs and miRs in human retinal endothelial cells (HRECs). About 25 mmol/L (HG) glucose caused downregulations of (a‐c) SIRT3, SIRT4, and SIRT5 in HRECs compared to 5 mmol/L glucose (NG), but not 25 mmol/L L‐glucose (osmotic control, OSM). Such downregulations were prevented by resveratrol treatment. (d‐f) miR1, miR19b, and miR320a were upregulated when exposed to HG compared to NG and such upregulations could not be prevented by resveratrol (Resv = resveratrol, * = significantly different compared to NG, ξ = significantly different from HG; miRNA levels are expressed as a ratio of U6 snRNA (U6); mRNAs expressed as a ratio to β‐actin; *n* = 6/group)

**Figure 2:**
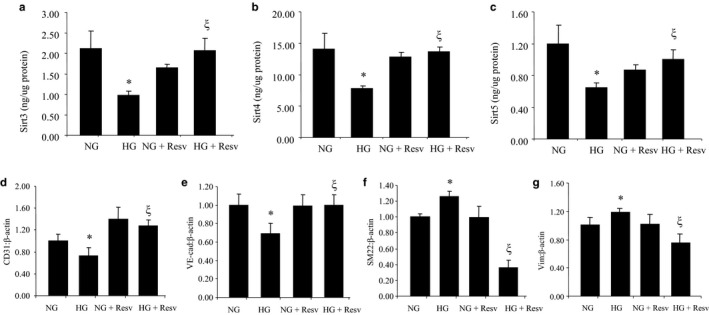
Protein expressions of SIRTs and mRNA expressions of endothelial and mesenchymal markers in HRECs. (a‐c) Expression levels of SIRT3, SIRT4, and SIRT5 proteins by ELISA showed downregulation of these markers in HG‐treated HRECs compared to NG‐treated HRECs. Treatment with resveratrol prevented such downregulations. (d‐g) When exposed to HG, expression of endothelial markers (CD31, VE‐cad) were downregulated and mesenchymal markers (SM22, Vim) were upregulated by real‐time qRT‐PCR compared to NG. Treatment with resveratrol prevented such changes (NG = 5 mmol/L glucose, HG = 25 mmol/L glucose, Resv = resveratrol, * = significantly different from NG, ξ = significantly different from HG, mRNAs expressed as a ratio to β‐actin; *n* = 6/group; VE‐cad, VE cadherin; Vim, vimentin)

In our previous duration‐dependent studies, we have established that 48 hr of HG exposure to retinal endothelial cells can significantly alter several vasoactive factors and extracellular matrix proteins when compared to cells cultured in 5 mmol/L glucose (NG) (Biswas et al., 2018). Hence, for the current study, we selected this time point for our subsequent experiments. HG exposure for 48 hr markedly reduced the mRNA expressions of all mitochondrial SIRTs (Figure 1). No such changes were seen following incubation with 25 mmol/L L‐glucose (osmotic control). We further measured protein levels of these SIRTs using ELISA. In keeping with mRNA levels, protein levels of SIRTs 3, 4, and 5 were also significantly reduced under HG environments (Figure 2).

**Figure 3:**
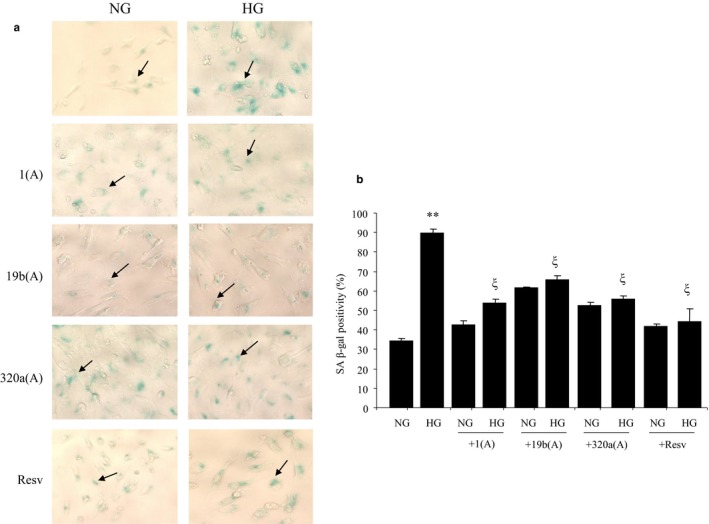
Transfection of miRNA antagomirs inhibited the glucose‐induced, SIRT‐mediated, cellular senescence in HRECs. (a) Senescence‐associated staining (SA β‐gal) of HRECs in HG and NG following treatment with miR1, miR19b, and miR320a antagomir or resveratrol treatment. (b) Quantification of SA β‐gal positivity. HG‐induced β‐gal positivity was prevented by resveratrol and transfection with miR1, miR19b, and miR320a antagomirs (NG = 5 mmol/L glucose, HG = 25 mmol/L glucose, Scr = scramble, 1(A) = miR1 antagomir, 19b(A) = miR19b antagomir, 320a(A) = miR320 antagomir, Resv = resveratrol, ** *p* < .01 versus NG; ξ *p* < .01 versus HG. Original magnification 200× for all microscopies, arrow = SA β‐gal positive)

To understand the biological significance of such reductions, we examined cellular senescence. To this extent, we stained the cells for SA β‐gal, which is a well‐established and well‐characterized assay to examine cellular senescence (Debacq‐Chainiaux et al., 2009; Mortuza et al., 2013). As hypothesized, HG caused notable increases in SA β‐gal staining (Figure 3), further confirming our previous report of glucose‐induced acceleration of EC senescence (Mortuza et al., 2013).

To understand a cause–effect relationship, we used a chemical stimulator of SIRTs (i.e., resveratrol). Resveratrol treatment not only prevented HG‐induced reductions in mitochondrial SIRTs, but it also prevented glucose‐induced accelerated aging in the EC (Figures 1, 2, 3).

To further examine glucose‐induced cellular damage, we examined a specific cellular phenotypic alteration induced by glucose, namely endothelial‐to‐mesenchymal transition (EndMT). We and others have previously demonstrated this phenomenon in glucose‐induced cellular damage (Feng et al., 2016). In this study, we found that glucose caused EndMT in the ECs, as evidenced by reduced endothelial markers (CD31 and VE cadherin) and increased mesenchymal markers (SM22 and vimentin). Remarkably, the use of resveratrol prevented such changes (Figure 2).

As the previous sets of experiments indicated that these mitochondrial SIRTs are possibly of pathogenetic significance in the context of chronic diabetic complications, we further explored the regulations of these molecules. Particularly, we explored whether specific microRNAs (miRNAs, miRs) regulate such mitochondrial SIRTs. Based on our previous array analyses (McArthur, Feng, Wu, Chen, & Chakrabarti, 2011) and using open‐sourced software (Targetscan, microRNA.org), we identified three potential candidates for such analyses. These miRs include miR‐1 (targets SIRT3), miR‐19b (targets SIRT4), and miR‐320a (targets SIRT5). We first confirmed the array data using qRT‐PCR. Such analyses showed that HG caused the upregulation of these miRs (Figure 1). Following confirmation, we then proceeded to establish a direct relationship between these miRs and the SIRTs under investigation in our subsequent experiments.

**Figure 4:**
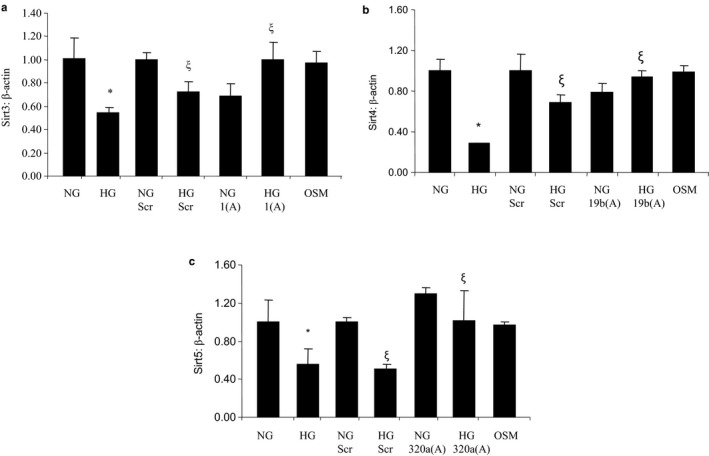
mRNA expressions of mitochondrial SIRTs by qRT‐PCR following transfection with miRNA antagomirs in HRECs. (a‐c) Transfection of HRECs with miR‐1, miR‐19b, and miR‐320a antagomirs prevented HG‐induced downregulations of SIRT3, SIRT4, and SIRT5. But no effects on SIRT expressions were seen when treated with scrambled or 25 mM L‐glucose (OSM) (NG = 5 mmol/L glucose, HG = 25 mmol/L glucose, Scr = scramble, 1(A) = miR1 antagomir, 19b(A) = miR19b antagomir, 320a(A) = miR320 antagomir, * = significantly different from NG, ξ = significantly different from HG; mRNAs expressed as a ratio to β‐actin; *n* = 6/group. Each experiment was performed three times independently)

For such analyses, we used both chemical and molecular approaches. We used resveratrol and miRNA antagonists (antagomirs). Resveratrol was effective in preventing glucose‐induced downregulations of SIRTs without affecting their corresponding miRNAs (Figure 1). Next, we performed antagomir transfections for miRNAs 1, 19b, and 320a, as these miRNAs were found to be upregulated by HG. Cells transfected with specific antagomirs (miR‐1 for SIRT3, miR‐19b for SIRT4, and miR‐320a for SIRT5) prevented HG‐induced downregulations of specific SIRTs (Figure 4). Along with the correction of HG‐induced SIRT alterations, such transfections additionally prevented glucose‐induced cellular senescence as evidenced by SA β‐gal staining with variable efficiency (Figure 3). Furthermore, using western blotting, Sirt3 protein levels decreased after glucose treatment, however, this reduction was prevented by miRNA antagomirs. Detection of lysine‐containing residues indicated that the acetylation level was increased and prevented by transfection of miRs, 1, 320a, and 19b antagomirs (Figure S1).

**Figure 5:**
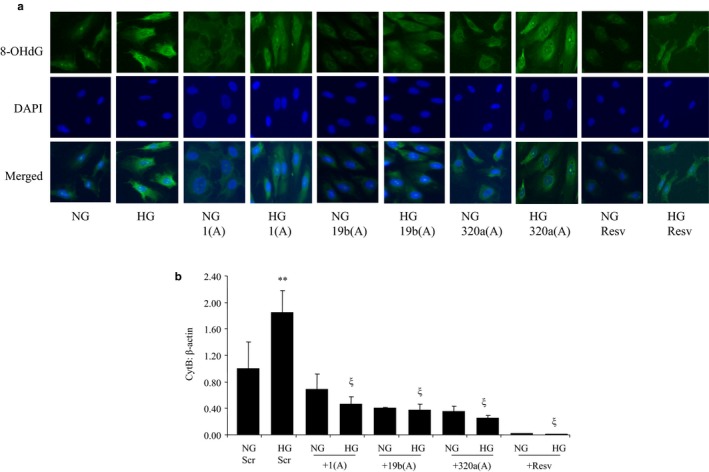
Oxidative stress induced by high glucose was prevented with miR antagomir transfection and resveratrol treatment in HRECs. (a) 8‐OHdG immunofluorescence staining of HRECs in HG and NG followed by treatment with miR‐1, miR‐19b, and miR‐320a antagomirs or resveratrol. (b) mRNA expressions of cytochrome B (CytB) were upregulated by HG and reversed by transfection with miRNA antagomirs and resveratrol treatment (NG = 5mmol/L glucose, HG = 25mmol/L glucose, Scr = scramble, 1(A) = miR1 antagomir, 19b(A) = miR19b antagomir, 320a(A) = miR320 antagomir, Resv = resveratrol, **p < .01 versus NG; ξ significant difference compared to HG, 400× magnification)

As mitochondrial oxidative stress is a key initiating event in glucose‐induced cellular damage, we used 8‐OHdG as a marker of oxidative DNA damage. We and others have used this sensitive and specific approach previously (Farhangkhoee et al., 2006; La Sala et al., 2015). Indeed, glucose caused oxidative DNA damage, as evidenced by increased nuclear staining. Such damages were prevented by resveratrol, as well as by the three antagomirs with variable efficiencies. In addition, we assessed Cytochrome B (CytB) expressions as a second marker of mitochondrial damage (Duraisamy, Mohammad, & Kowluru, 2019). Glucose caused significant upregulations of CytB mRNA expressions (Figure 5). Interestingly, specific antagomir transfection or resveratrol prevented such upregulations. To measure the effects of glucose on mitochondrial function, JC‐1 staining was performed for the mitochondrial potential (Δ_ψm_). The results demonstrated that HG decreases the mitochondrial potential and these decreases can be prevented by the transfection of miRNA antagomirs (Figure S2).

**Figure 6:**
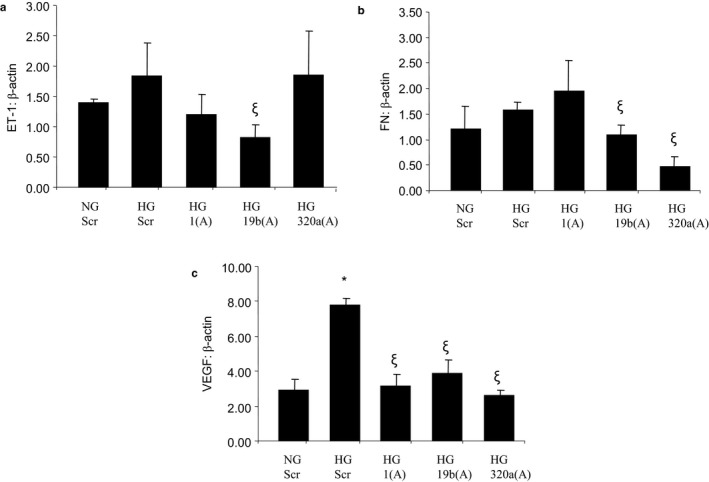
Expressions of (a) ET‐1, (b) FN, and (c) VEGF following transfection with miRNA antagomirs in HRECs. ET‐1 mRNA upregulation was prevented by miR‐1 and miR‐19b antagomirs. However, miR‐1 antagomir was not effective in preventing HG‐induced FN upregulation. HG‐induced VEGF upregulations were prevented by all antagomirs (NG = 5mmol/L glucose, HG = 25mmol/L glucose, Scr = scramble, 1(A) = miR1 antagomir, 19b(A) = miR19b antagomir, 320a(A) = miR320 antagomir, * = significantly different from NG, ξ = significantly different from HG; mRNAs expressed as a ratio to β‐actin; *n* = 6/group)

Finally, we examined specific effects of these SIRTs and their regulation by specific miRNAs on aberrant glucose‐induced macromolecule productions in cells. To this extent, we investigated endothelin‐1 (ET‐1), vascular endothelial growth factor (VEGF), and fibronectin (FN) because these molecules have been extensively studied in a diabetic environment and their glucose‐induced upregulations were confirmed by our laboratory and others (Chen et al., 2010; Hsu, Yin, & Tian, 2005; Padilla et al., 2018). As expected, HG significantly upregulated the three macromolecules. Conversely, glucose‐induced upregulations of ET‐1, VEGF, and FN were impacted by the three miRNA antagomirs at variable efficiencies. For example, although miR‐1 and miR‐19b antagomirs were effective in preventing glucose‐induced ET‐1 upregulations, miR‐320a antagomir failed to do so. On the other hand, miR‐19b and miR‐320a antagomirs, but not miR‐1 antagomir, were effective in preventing glucose‐induced FN upregulation; while, VEGF upregulations were prevented by all three antagomirs (Figure 6).

**Figure 7:**
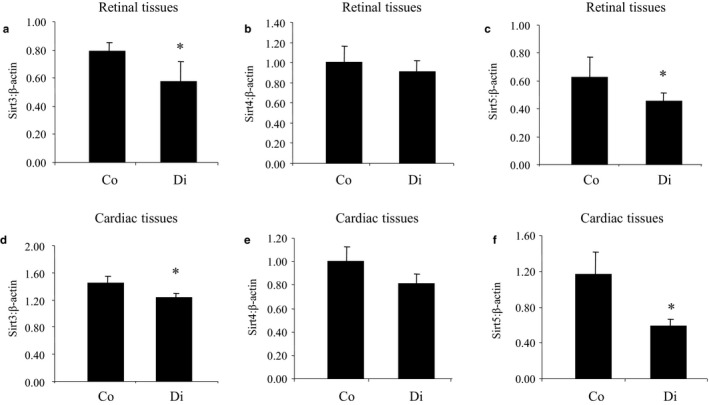
Expression of mitochondrial SIRT mRNAs in the tissues from diabetic animals. Using qRT‐PCR analyses, mRNA levels of SIRT3, SIRT4, and SIRT5 are downregulated in diabetes. (a‐c) Retinal and (d‐f) cardiac tissues of diabetic animals compared to age‐ and sex‐matched controls (Co = control, Di = Diabetic, mRNA levels are expressed as a ratio to β‐actin. * = significantly different from the control group, *n* = 8/group)

As a proof of principle, we further expanded our study to investigate whether similar reduction of mitochondrial SIRTs occurs in the tissues affected by chronic diabetic complications. To this extent, we examined two organs affected by chronic diabetic complications and are of interest to us. Particularly, we examined retinal and cardiac tissues from STZ‐induced diabetic mice after 2 months of follow‐up. These diabetic mice showed hyperglycemia, polyuria, and reduced body weight gain (data not shown) compared to their nondiabetic counterparts (receiving buffer injection only). Two months of poorly controlled diabetes (maintained without exogenous insulin) significantly reduced Sirt3 and Sirt5 mRNA expressions in retinal and cardiac tissues (Figure 7), while Sirt4 expressions were also decreased, but are not statistically significant.

## 4 DISCUSSION

In this study, we have demonstrated that high glucose causes oxidative stress, DNA damage and cellular aging, as well as EndMT in retinal EC. Such glucose‐induced cellular damage is mediated, at least in part, through reduced production of mitochondrial SIRTs, namely SIRTs 3,4, and 5. We have further demonstrated the regulation of these SIRTs through specific miRNAs and that similar changes exist in the retina and hearts of chronically diabetic animals.

Glucose‐induced oxidative damage is a well‐established phenomenon in the initiation and progression of chronic diabetic complications (Chen et al., 2010; Feng et al., 2016; Mortuza et al., 2013). The findings on oxidative damage from our study are in keeping with previous demonstrations by others and us (Chen et al., 2010; La Sala et al., 2015). The novel aspects of this study are the demonstration of mitochondrial SIRTs and their regulation by specific miRs in mediating such effects. One of the intriguing findings is that resveratrol treatment prevented glucose‐induced DNA damage, as evidenced by the 8‐OHdG stain, and it prevented glucose‐induced CytB upregulation. Furthermore, the effect of resveratrol was independent of miRNAs. On the other hand, several miRNAs targeting specific SIRTs were able to do this with variable efficiencies. Exact reasons for such discrepancies are not clear. Possible speculative mechanisms may include involvement of epigenetic pathways including additional effects of miRNAs. Additional investigation(s) will be needed to decipher such processes.

We used a well‐established in vitro model system, using human retinal microvascular endothelial cells, in order to demonstrate the glucose‐mediated mechanisms of SIRTs. HRECs are a well‐studied cell line and have been extensively used by us and others for such studies (Biswas et al., 2018; Qiu et al., 2018). In addition to HRECs, we also used STZ‐induced diabetic mice in our study. This model lends itself to study early molecular and cellular changes in diabetic retinopathy and other chronic diabetic complications (Biswas et al., 2018; Feng et al., 2016; Wang et al., 2016).

SIRTs 3,4, and 5 are located in the mitochondrial matrix and act by deacetylating lysine residues. As reversible lysine acetylation is a major mechanism of posttranslational modifications for proteins, which occurs through the alteration of charges on lysine residues, SIRTs may influence this acetylation process and consequently impact a large number of proteins that regulate multiple cellular processes (Gertz & Steegborn, 2016). SIRTs have also been implicated in multiple disease processes (Osborne, Bentley, Montgomery, & Turner, 2016). Moreover, among the SIRTs, SIRT3 is relatively well studied. Of relevance to our study, SIRT3 has been shown to play a pathogenetic role in oxidative stress‐mediated retinal cell death and retinal dysfunction (along with SIRT5) (Liu, Cao, Xu, & Li, 2015; Luo et al., 2017). Furthermore, in the cardiac tissues of diabetic rats, resveratrol has been shown to improve mitochondrial oxidative phosphorylation mediated by SIRT3 (Bagul, Katare, Bugga, Dinda, & Banerjee, 2018). All three mitochondrial SIRTs demonstrate deacetylase activity. In addition to deacetylation, SIRT3 has been shown to possess decrotonylase activity; whereas, SIRT 4 demonstrates ADP‐ribosyltransferase and lipomidase activities and SIRT5 further demonstrates desuccinilase, degluterylase and demalonylase properties (Singh et al., 2018). With this large array of protein modifying actions, it is conceivable that SIRT alterations play critical roles in multiple disease processes. Indeed, the pathogenetic role of mitochondrial SIRTs has been shown in diabetes, aging, neurodegenerative disorders, and multiple malignancies (Jęśko, Wencel, Strosznajder, & Strosznajder, 2017).

Although the mechanistic roles of mitochondrial SIRTs have not been well studied in the context of chronic diabetic complications, we have previously demonstrated a potential role for SIRT1 in diabetic retinopathy (Mortuza, Feng, & Chakrabarti, 2014). In particular, we have confirmed the miR‐195‐mediated regulations of SIRT1 (Mortuza et al., 2014). Undoubtedly, as miRNA‐based studies emerge, novel functions of miRNAs will continue to be revealed. Our study shows that specific miRs also regulate the expressions of mitochondrial SIRTs. All three miRs examined in this study have been looked at in some chronic diabetic complications by us and others (Feng et al., 2014; He et al., 2019; Yildirim, Akman, Catalucci, & Turan, 2013) and were found to play pathogenetic roles in specific organ damage (He et al., 2019). In this study, we have shown that these miRs regulate some of the vasoactive factors and the production of ECM proteins. Although these marcomolecules are not direct targets of the miRNAs under study, it may be possible that SIRTs mediate these molecules through other molecular mechanisms. However, such notion needs to be further established by additional mechanistic studies. As one miR may regulate multiple mRNAs and one mRNA may be regulated by multiple miRs, it appears that an intricate regulatory relationship exists between glucose‐induced oxidative stress, miR production, epigenetic mechanisms (such as acetylation and deacetylation), and other additional mechanisms regulating gene expression (Feng, Ruiz, & Chakrabarti, 2013; Reddy & Natarajan, 2011). Better understanding of such regulatory mechanisms is extremely important to develop targeted treatment for diabetic retinopathy and other chronic diabetic complications.

## CONFLICTS OF INTEREST

No conflicts of interest, financial or otherwise, are declared by the author(s).

## AUTHOR CONTRIBUTIONS

JL performed the experiments and wrote/edited manuscript, S Chen performed the experiments, SB performed the experiments and helped with data analysis and manuscript writing/editing, NN performed the experiments, YC contributed to discussion and wrote/edited manuscript. SC performed the experiments, contributed to discussion and wrote reviewed/edited the manuscript. BF contributed to performed the experiments, discussion and wrote/edited manuscript.
