# Supplementary figures and images for: Glucose‐induced oxidative stress and accelerated aging in endothelial cells are mediated by the depletion of mitochondrial SIRTs
Source: Physiol Rep. 2020 Feb 5;8(3):e14331. doi: 10.14814/phy2.14331 (PMC7002531; doi:10.14814/phy2.14331)

Suppl. Fig. 1

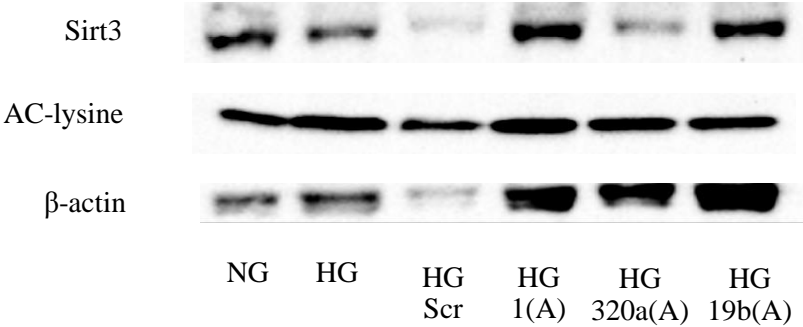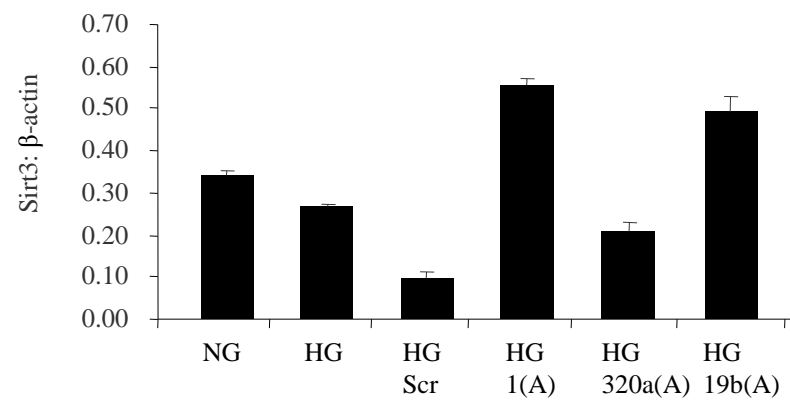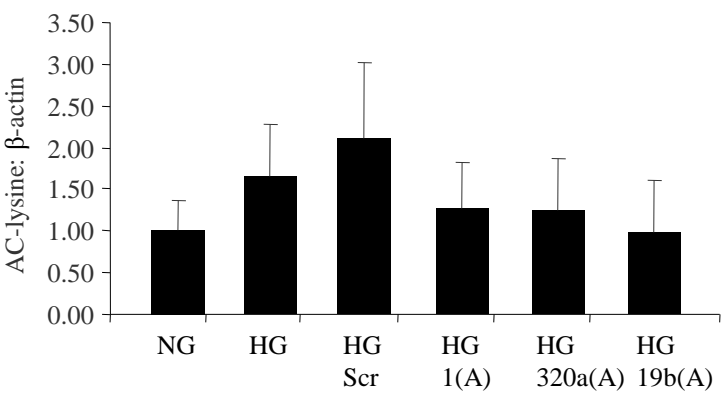

Suppl. Fig. 2

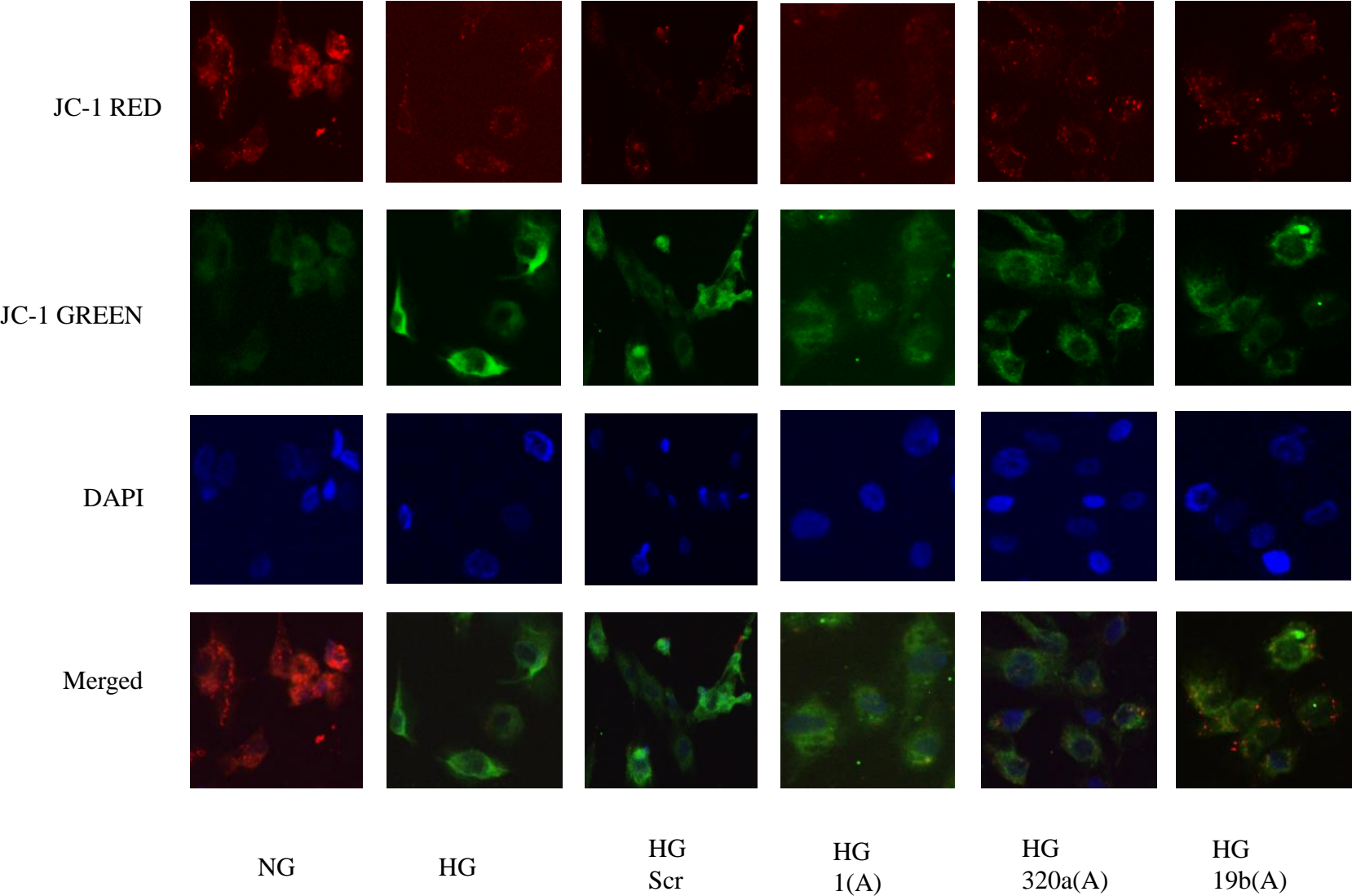

Supplement: Supplementary file 1 [file PHY2-8-e14331-s001.pdf]
